# Overexpression of *SlUPA-like* induces cell enlargement, aberrant development and low stress tolerance through phytohormonal pathway in tomato

**DOI:** 10.1038/srep23818

**Published:** 2016-03-30

**Authors:** Baolu Cui, Zongli Hu, Jingtao Hu, Yanjie Zhang, Wencheng Yin, Zhiguo Zhu, Ye Feng, Guoping Chen

**Affiliations:** 1 Key Laboratory of Biorheological Science and Technology (Chongqing University), Ministry of Education, Bioengineering College, Chongqing University, Chongqing 400044, People’s Republic of China

## Abstract

*upa20* induces cell enlargement and hypertrophy development. In our research, overexpression of *SlUPA-like*, orthologous to *upa20*, severely affected the growth of vegetative and reproductive tissues. Wilted leaves curled upwardly and sterile flowers were found in transgenic lines. Through anatomical analysis, palisade and spongy tissues showed fluffy and hypertrophic development in transgenic plants. Gene expression analysis showed that GA responsive, biosynthetic and signal transduction genes (e.g. *GAST1*, *SlGA20OXs, SlGA3OXs*, *SlGID1s*, and *SlPREs*) were significantly upregulated, indicating that GA response is stimulated by overproduction of *SlUPA-like*. Furthermore, *SlUPA-like* was strongly induced by exogenous JA and wounding. Decreased expression of *PI-I* and induced expression of *SlJAZs* (including *SlJAZ2*, *SlJAZ10* and *SlJAZ11*) were observed in transgenic plants, suggesting that JA response is repressed. In addition, *SlUPA-like* overexpressed plant exhibited more opened stoma and higher water loss than the control when treated with dehydration stress, which was related to decreased ABA biosynthesis, signal transduction and response. Particularly, abnormal developments of transgenic plants promote the plant susceptibility to *Xanthomonas campestris pv. campestris*. Therefore, it is deduced from these results that *SlUPA-like* plays vital role in regulation of plant development and stress tolerance through GA, JA and ABA pathways.

*Xanthomonas* causes multiple of crop diseases, such as vascular wilts, cankers, leaf spots, fruit spots and blights. The susceptible plant often display pustules on the abaxial surface of leaf, such as the enlargement of mesophyll cell^1^. Palisade and spongy parenchyma cells are strongly enlarged along with the decreased starch contents in chloroplasts, which is similar to the phenotypes induced by AvrBs3^2^. The AvrBs3 family proteins are type III effectors delivered by *Xanthomonas*. The N-terminal and C-terminal portion of AvrBs3 are highly conserved and their biological activities are related to the particular repetitive region. The conserved C-terminal contains potent nuclear localization signal (NLS). So, AvrBs3 like plant transcription factor locates to the nuclear and modulates the transcriptional expressions of host genes^3^. Marois, *et al.*^1^ reported that 22 induced and 2 repressed genes are identified, of which 13 genes are confirmed to be induced by *X. campestris* pv. *vesicatoria* (*Xcv*). The 13 genes were designed as *upa1-13*(up-regulated by AvrBs3), which show high homology to auxin-induced genes, expansion-like genes and pectate lyase genes. Among them, a few gene sequences exhibit low homology to gibberellins (GA)-responsive genes^1^. Therefore, the specific host-gene activation by AvrBs3 is a complex signaling network, of which the function of phytohormones is indispensable.

Phytohormones regulate plant growth and development, but their signaling pathways are also captured by pathogens to defend against plant defenses. Some effectors or phytotoxins secreted by pathogens have evolved to overcome pattern triggered immunity (PTI) of plants and establish the success infections by means of reprogramming plant hormonal pathway^4^. Coronatine (COR) produced by *Pseudomonas syringae*, suppresses the SA-mediated defense in planta^5^,^6^. Hopl1, one of the effectors of *P. syringae*, disrupts HSP70 (HEAT SHOCK PROTEIN70) in chloroplasts and alters the biosynthesis of SA^7^,^8^. Two effectors secreted by *Xanthomonas*, XopJ and XopD, suppress SA-dependent defense^9^,^10^. Effectors also use the antagonistic effects between JA and SA to colonize host plants. HopX1 from *Pseudomonas syringae*, promotes the degradation of jasmonate ZIM-domain (JAZ) proteins through its central ZIM domain. The JA-induced defenses are activated to counteract SA-dependent defenses^11^. In general, SA-mediated signaling pathway are involved in resistance to biotrophic pathogens and activation of JA response leads to defense against necrotrophic pathogens^12^. In addition, a balance of both JA and GA sustains the antagonistic behavior of defense and plant growth. JA is associated with activation of defense against pathogen ingress and GA promotes plant growth at the expense of defense^13^. GA was firstly identified in the pathogenic fungus *Gibberella fujikuroi*, the causal agent of excessive elongation of infected plant. Knockout or overexpression of *Elongated uppermost internode* (*Eui*) genes encoding a GA deactivating enzyme was susceptible and resistant to the infection of *Xanthomonas* and *Magnaporthe oryzae* in rice^14^. However, no information is obtained so far that the effector proteins secreted by pathogens directly interfere with the GA signaling pathway.

Stomata are important entry sites for pathogens ingress. Pathogens have evolved effector proteins or phytotoxins to counteract host stomatal defenses by inhibiting stomatal closure or promoting stomatal opening^15^. The phytotoxin COR can impede pathogen-associated molecular pattern (PAMP)-induced stomatal closure by the ABA-dependent pathway^16^. HopX1 from virulent Pta11528 bacteria play an important role in maintaining stomatal aperture^11^. Likewise, expression of HopF2 results in significantly wider stomatal aperture and insensitivity to PAMP-induced stomatal closure^17^. Besides, it was reported that activation of ABA signaling pathway could inhibit stomatal opening induced by pathogens. Overexpression of RCAR3, one of ABA receptors, inhibits stomatal reopening during *Pst* DC3000 infection^18^. Pst DC3000-induced stomatal closure is affected by the mutation of *NCED1* gene (*notabilis* (*not*) mutant)^19^. Whether *Xanthomonas* or AvrBs3 acts on stomatal immunity still needs more evidence.

It is reported that bHLH family gene participates in regulation of GA and JA metabolisms or responses. *PHYTOCHROME-INTERACTING FACTOR3-LIKE5* (*PIL5*) inhibits seed germination by repressing the expressions of GA biosynthetic genes in Arabidopsis^20^. Phytochrome interacting factors (PIFs) control hypocotyl elongation in Arabidopsis seedlings^21^. MYCs (MYC2, MYC3 and MYC4) act redundantly on the activation of JA response^22^. But, *upa20*, anther bHLH family gene, is not proved to be implicated in alteration of GA or JA response.

*upa20* is the direct target of AvrBs3 in pepper. Transient expression of *upa20* induces hypertrophy phenotypes and decreased starch contents in chloroplasts. Hypertrophy of mesophyll cells is attributed to enlargement of cell size, whereas cell division in hypertrophic leaves is not mentioned^2^. There are two homologous genes of *upa20* in tobacco and one in tomato genomes^23^. Overexpression of *Nt upp-L* results in hyperplasia development. Palisade and spongy parenchyma cells of transgenic leaves are smaller but abundant in number compared with control. The molecular mechanism of hyperplasia development is due to acceleration of cell cycle from G1 phase to mitotic phase, leading to the promotion of cell division^23^. From these results, *upa20* and *upp-L* in pepper and tobacco participate in different regulatory mechanism. The function of homologous gene is still undetermined in tomato.

Inducing visible cellular changes in transient expression of *avrBs3* selects the scope of the AvrBs3-specific effects to *solanaceous* plants, of which the tomato is selected as our research material. The function of *SlUPA-like*, homologous gene to Ca *upa20*, was identified in tomato. Constitutive expression of *SlUPA-like* induced cell enlargement and reduced cell numbers in leaves of wild type tomato, Ailsa Craig (AC^++^). Moreover, overexpression of *SlUPA-like* improved GA response and susceptibility to pathogen infection together with repressed JA and ABA response.

## Results

### Cloning and molecular characterization of *SlUPA-like*

In four distinct subfamilies of Upp/Upa-like transcription factors by the phylogenetic analysis, the gene Sl AW034575 from the group 1of *solanaceous* plants is orthologous to Ca *upa20*, whereas not to Nt *UPP-L*^23^. We cloned the full-length cDNA of the Sl AW034575 gene from the AC^++^ by rapid amplification of cDNA ends (RACE) and named it *SlUPA-like* following the nomenclature of *upa* (upregulated by AvrBs3). The putative SlUPA-like protein has 330 amino acids with a typical bHLH domain region from amino acid 156 to 214, and an estimated molecular mass of 36.3 kD. Additionally, we searched DNA sequence of *SlUPA-like* from tomato genome (GenBank accession No.NP_001266190.1). Alignment of the genomic DNA and cDNA showed that the *SlUPA-like* contained seven exons and six introns.

### The expression profiles of *SlUPA-like* in different tissues of wild type

**Figure 1:**
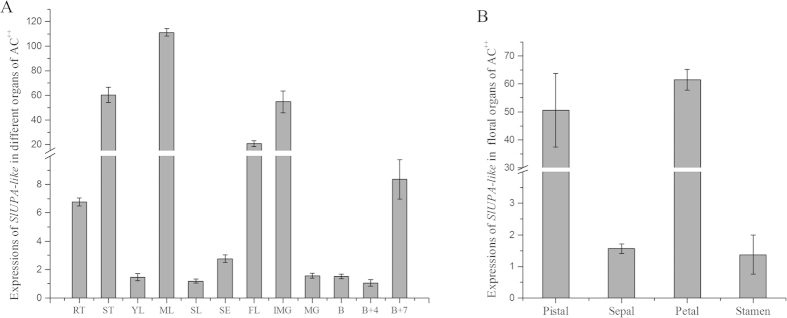
Expression profiles of *SlUPA-like* in different tissues of AC^++^. The expression level of *SlUPA-like* in different tissue of AC^++^ (**A**) and different organ of flower (**B**). RT, roots; ST, stems; YL, young leaves; ML, mature leaves; SL, senescent leaves; SE, sepals of flower in anthesis; FL, flower; IMG, immature green fruits; MG, mature green fruits; B, breaker fruits; B + 4, 4 d after breaker fruits; B + 7, 7 d after breaker fruits.

To clarify the role of *SlUPA-like* in plant development, its transcript accumulation in various tissues was quantified by quantitative RT-PCR. As shown in Fig. 1A, *SlUPA-like* was high expressed in stem, mature leaves, flowers and immature fruits, whereas low in root, young leaves, senescent leaves, sepals, and ripe fruits. Besides, pistils and petals showed high *SlUPA-like* accumulation compared to sepals and stamens in flower tissues (Fig. 1B). These results implied that *SlUPA-like* is expressed in specific organ that grows larger via cell elongation or enlargement.

### Overexpression of *SlUPA-like* induces cell enlargement by regulation of cell cycle

**Figure 2:**
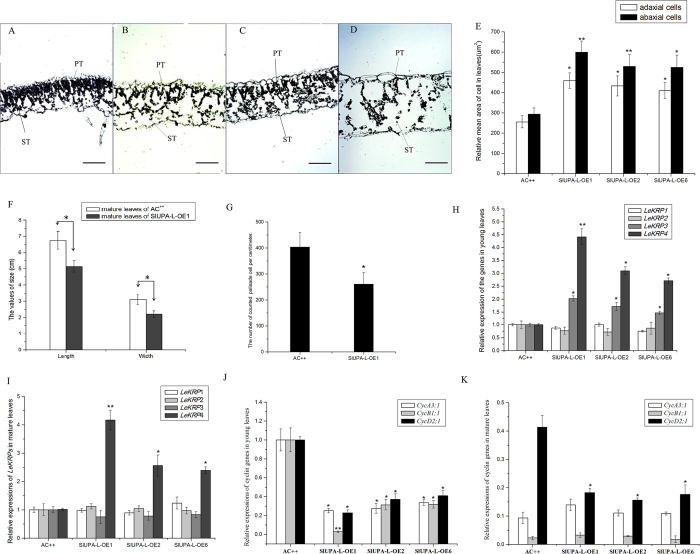
Cell enlargement induced by *SlUPA-like* and expression analysis of *LeKRPs* and cycle related genes in leaves of transgenic lines and control. Light micrographs of cross sections of young leaves of AC^++^ (**A**), young leaves of *SlUPA-L*-OE plants (**B**), mature leaves of AC^++^ (**C**), and mature leaves of *SlUPA-L*-OE plants (**D**), magnification, 80×. PT, palisade tissue; ST, spongy tissue. (**E**).The relative average area of adaxial and abaxial cells from control and *SlUPA-L*-OE leaves were calculated. (**F**). Statistical analysis of length and width of the first leaflets. (**G**). Calculated number of palisade cell per centimeter from the first leaflets. Expression analysis of *LeKRPs* both in young leaves (**H**) and mature leaves (**I**). Expression analysis of cell cycle related genes both in young leaves (**J**) and mature leaves (**K**). Each value represents the mean ± SD of three replicates. Asterisks indicate a significant difference (**P < 0.01 and *P < 0.05) between WT and transgenic lines.

To further analyze the biological function of *SlUPA-like* in tomato, we generated transgenic tomato plant by overexpressing *SlUPA-like*. A total of 8 independent transgenic lines were obtained and three of them were selected for further characterization. From Fig. 2A–D, the leaves of *SlUPA-like*-overexpressed lines (*SlUPA-L*-OE) showed fluffy and hypertrophic development in both palisade and spongy parenchyma tissues, which leads to thickened blades. From the anatomical analysis, palisade tissue was mostly destroyed in mature leaves in contrast to relative integrity in young leaves of transgenic lines. Moreover, the mean area of adaxial and abaxial pavement cells were simultaneously enlarged in leaves of transgenic lines (Fig. 2E, Fig. S1). However, the length and width of the first leaflet were both significantly reduced compared to the control (Fig. 2F), although the ratio of length to width was almost unaffected (data not shown), indicating that cell numbers in *SlUPA-L*-OE leaves were decreased. Likewise, the number of palisade cell per centimeter was decreased in transgenic plants (Fig. 2G). The hypertrophy development is due to cell enlargement, which results from the abnormal expressions of E2F-response genes^1^,^23^. In Arabidopsis, over-expressions of *KRP1* and *KRP2* lead to a compensatory increase in cell volume in response to severely reduced cell number and expressing of *KRP1* and *KRP4*, driven by *AtML1* promoter, induces leaves curling upward^24^,^25^. In this assay, expression levels of cyclin-dependent kinase inhibitor genes *KIP-RELATED PROTEIN1/INHIBITOR1 OF CDC2 KINASE* (*LeKRPs*) were determined by RT-PCR^26^. Figure 2H showed that *LeKRP3* and *LeKRP4* were significantly induced in young leaves of transgenic plants, especially *LeKRP4* that is orthologous to the *AtKRP1* (Fig. S2). In mature leaves of transgenic plants, *LeKRP4* level was still higher than control (Fig. 2I). Besides, expression level of three cyclin genes were investigated^27^. *CycA3;1*, *CycB1;1* and *CycD2;1* were significantly repressed in young leaves of transgenic plants as well as *CycD2;1* in mature leaves (Fig. 2J,K), indicating that overexpression of *SlUPA-like* represses the progress of cell cycle and promotes cell enlargement. Therefore, these results revealed that overexpression of *SlUPA-like* induces cell enlargement, which is due to the repressed progress of cell cycle.

### Overexpression of *SlUPA-like* interferes with the growth and development of plants

**Figure 3:**
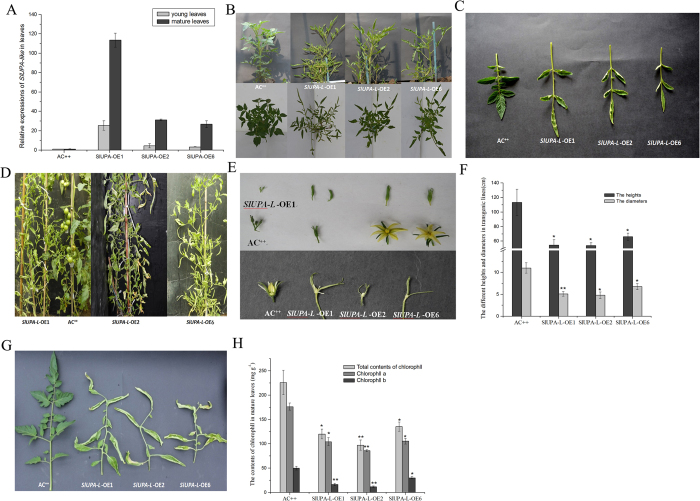
The aberrant developments showed in *SlUPA-L*-OE plants. (**A**). Expression analysis of SlUPA-like in young and mature leaves. The phenotypes of young plants (**B**), young leaves (**C**), and adult plants (**D**). (**E**). The flowers of transgenic plants (the top half of this picture) and opened flowers artificially (the bottom half of this picture). (**F**). The mean height and diameter in adult plants. The phenotypes (**G**) and chlorophyll content (**H**) of mature leaves of transgenic lines and wild type. Each value represents the mean ± SD of six replicates. Asterisks indicate a significant difference (**P < 0.01 and *P < 0.05) between WT and transgenic lines.

In addition to inducing the cell enlargement, overexpression of *SlUPA-like* severely interfered with plant growth and development. At seedling stage, the expression of *SlUPA-like* in transgenic lines was 2 to 25-fold higher than control (Fig. 3A). Compound leaves of *SlUPA-L*-OE lines bear sparse leaflets, especially fewer intercalary leaflets. All of the leaflets were curled upwardly, leading to the whole plant looked slender (Fig. 3B,C). In adult plants, the transcripts of *SlUPA-like* in mature leaves of transgenic lines were 20 to 120-folds higher than AC^++^ (Fig. 3A). We observed that *SlUPA-L*-OE lines cannot bloom and set up fruits (Fig. 3D). So, we could not get any seeds. When the flowers of transgenic plants were opened artificially, the petals, stamens and pistils were much smaller than control (Fig. 3E). The vegetative organs of transgenic lines also showed obvious alterations. The average height of transgenic lines was shorter than control as well as the average diameter (Fig. 3F). The average diameter of plants was measured at 10 cm above the ground. Furthermore, all mature leaves of transgenic lines were curled upwardly and wilted under the normal conditions (Fig. 3G), and the leaf vein was thicker and wider than wide type. From the cross-sectional view, the structure of vascular bundle in leaf veins was disrupted. Nearly all the cells were enlarged except a few phloem cells and even no apparent xylem cells were found (Fig. S3). Meanwhile, the contents of total chlorophyll, chlorophyll a and b were significantly reduced in mature leaves (Fig. 3H). Moreover, axillary buds furthest from the apex of plant could not form (Fig. S4), which resembles *pro* mutant carrying a point mutation in GRAS region^28^.

### Excessive accumulated transcripts of *SlUPA-like* promote GA response

**Figure 4:**
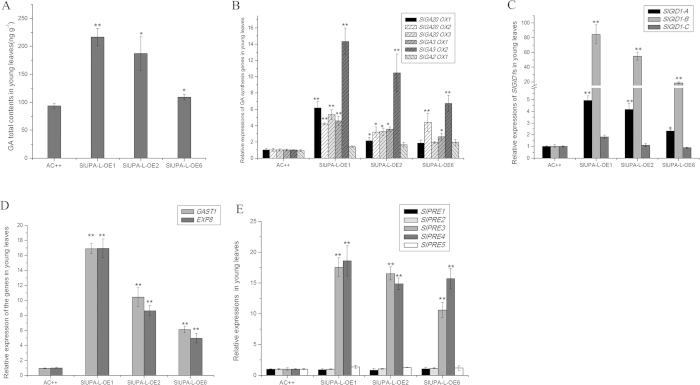
Expression analysis of GA biosynthetic and signal transduction genes along with detection of endogenous GA contents in young leaves. (**A**). Expression analysis of GA biosynthentic genes in young leaves. (**B**). Detection of endogenous GA contents in young leaves. Expression analysis of *SlGID1s* (**C**), GA responsive genes (**D**) and *SlPREs* (**E**) in young leaves. Each value represents the mean ± SD of three replicates. Asterisks indicate a significant difference (**P < 0.01 and *P < 0.05) between WT and transgenic lines.

Palisade and spongy parenchyma cells were strongly enlarged, which is similar to GA-induced phenotypes^28^. To determine the reason of hypertrophy phenotype, the endogenous content of GA in control and transgenic lines were investigated. Figure 4A showed that total GA contents in young leaves of transgenic lines were 1.2-to 2-fold higher than control. To reveal the molecular mechanism of GA accumulation in transgenic lines, the transcripts of GA biosynthetic and catabolic genes were examined. Figure 4B showed that expressions of GA synthetic genes were significantly increased, especially *SlGA3OX2*, whereas no obvious change was found in GA-deactivation gene *SlGA2OX1*. In tomato genome, there are three putative GA receptor genes, *GIBBERELLIN INSENSITIVE DWARF1s* (*GID1s*), which were tentatively termed: *SlGID1-A~C* (Fig. S5). Two *SlGID1s* were expressed at extremely high level in young leaves of the transgenic plants, especially *SlGID1-B* (Fig. 4C), indicating activating GA response may be due to the over-accumulated GA receptors. The abundance of *GAST1* mRNA (*gibberellic acid stimulated transcript 1*) is induced by GA_3_ treatment at transcriptional level^29^. EXP8 is proposed to induce cell expansion and *EXP8* expression is induced by exogenous GA^30^. In this assay, the transcriptional levels of both *GAST1* and *EXP8* in young leaves were significantly increased (Fig. 4D), indicating that overexpression of *SlUPA-like* promotes GA response. It was reported that increased GA contents and two *SlGID1s* gene expressions facilitate the formation of GA-GID1-DELLA protein complex, which downregulates the repression of DELLA proteins^31^. Therefore, hindering inhibitory effects of DELLA proteins may be one of strategies to enhance the GA response.

Paclobutrazol resistance1 (PRE1), a helix-loop-helix protein, alters various aspects of gibberellin-dependent response, such as elongation of petioles^32^. In tomato, there are five putative *PREs* that were tentatively termed: *SlPRE1~5* (Fig. S6). In our research, expression levels of two *SlPREs* in young leaves of transgenic lines were significantly higher than wild type (Fig. 4E), suggesting that over-accumulated transcripts of two *SlPREs* contribute to GA response.

**Figure 5:**
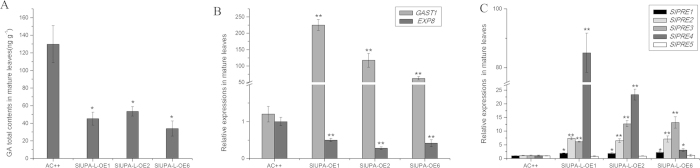
Expression analysis of GA biosynthetic and signal transduction genes along with detection of endogenous GA contents in mature leaves. (**A**). The detection of endogenous GA contents in mature leaves. Expression analysis of GA responsive genes (**B**) and *SlPREs* (**C**) in mature leaves. Each value represents the mean ± SD of three replicates. Asterisks indicate a significant difference (**P < 0.01 and *P < 0.05) between WT and transgenic lines.

Interestingly, endogenous GA contents in mature leaves of *SlUPA-L*-OE lines were reduced as well as the decreased expression of *EXP8* (Fig. 5A,B), whereas *GAST1* and four *SlPREs* (including *SlPRE1*-*SlPRE4*, particularly *SlPRE4*) were remarkably induced (Fig. 5B,C), suggesting that GA response was still stimulated in mature leaves.

### Overexpression of *SlUPA-like* represses JA response

**Figure 6:**
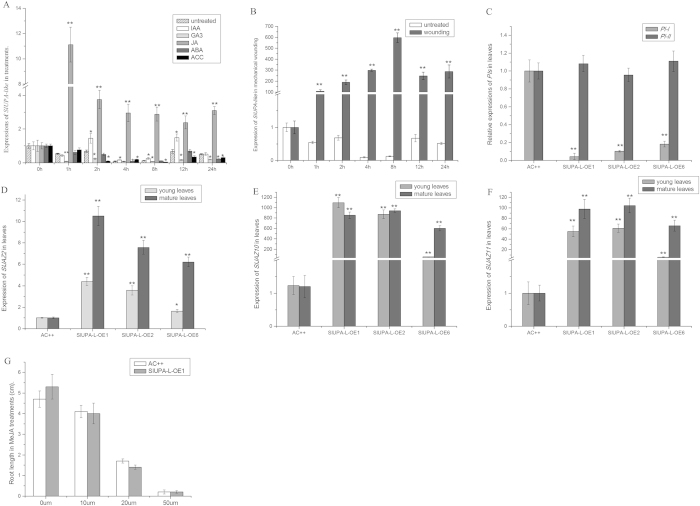
Overexpressions of *SlUPA-like* repressed JA response. Expression of *SlUPA-like* under phytohormonal treatments (**A**) and mechanical wounding treatments (**B**). (**C**). Expressions of two *PIs* in young and mature leaves of transgenic lines and control. Expressions of *SlJAZ2* (**D**)*, SlJAZ10* (**E**) and *SlJAZ11* (**F**) in young and mature leaves of transgenic lines and control. (**G**). Root growth treated with MeJA in AC^++^ and transgenic seedlings. Each value represents the mean ± SD of three replicates. Asterisks indicate a significant difference (**P < 0.01 and *P < 0.05) between WT and transgenic lines.

In order to characterize the function of *SlUPA-like* in hormonal response, its expression was investigated under five phytohormonal treatments. The transcripts of *SlUPA-like* were substantially accumulated within 1hour under JA treatment (Fig. 6A). JA also has an important role in wounding induction, which will rapidly increase within a few minutes^33^. Besides, the expressions of *JAZ* genes are simultaneously increased as primary responsive genes to JA treatment and mechanical wounding^34^. In our experiment, *SlUPA-like* expression was rapidly induced within 1 h and peaked at 8 h, followed by a decline but maintained at relatively high level until 24 h when treated with wounding (Fig. 6B), indicating that *SlUPA-like* is regarded as a primary responsive gene in response to JA. In transgenic plants, expression of *PI-I* was significantly repressed by 85~90% compared to control (Fig. 6C). It was reported that suppressed JA response is due to the JAZ proteins binding to MYC transcription factors^35^,^36^,^37^. In our experiment, *SlJAZ2*, *SlJAZ10* and *SlJAZ11* were severely induced in young and mature leaves of transgenic lines, especially *SlJAZ10* and *SlJAZ11* (Fig. 6D–F). Phylogenetic analysis showed that AtJAZ7, AtJAZ8, SlJAZ9, SlJAZ10 and SlJAZ11 belong to one clade and lack the conserved LPIAR motif, which makes AtJAZ8 stable in inhibition of JA response^38^. So, these results suggested that overexpression of *SlUPA-like* represses JA response by overexpressions of three *SlJAZ* genes. However, the inhibitory effects of three *SlJAZs* were limited. The length of root was repressed by exogenous MeJA in dose-dependent manner, which was similar to wild type (Fig. 6G). Therefore, the repressed JA response induced by overexpression of *SlUPA-like* can be overpassed by the application of exogenous JA, which may be due to the functional redundancy between *SlJAZs*.

### Overexpression of *SlUPA-like* stimulates stomata opening under dehydration stress

**Figure 7:**
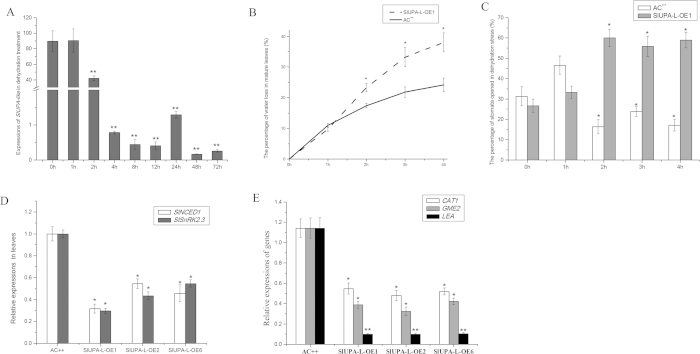
The dehydration treatments and related stomatal behavior in plants. (**A**). Expression analysis of *SlUPA-like* under dehydration stress. The percent of water loss (**B**) and the percentage of opened stomata in mature leaves (**C**) of control and *SlUPA-L*-OE1 under dehydration stress. Expression analysis of ABA signal transduction (**D**) and responsive genes in mature leaves. Each value represents the mean ± SD of three replicates. Asterisks indicate a significant difference (**P < 0.01 and *P < 0.05) between WT and transgenic lines.

Stomata controls gas exchange between leaves and environment along with water loss during transpiration. It is known that wide stomatal aperture leads to high water loss. In wild type plants, *SlUPA-like* was downregulated after 4 hours of dehydration treatment (Fig. 7A). Moreover, the leaves of *SlUPA-L*-OE lines exhibited higher water loss than control under dehydration stress (Fig. 7B). In general, stomatal behavior is the vital index for assessing water loss. Under dehydration stress, the percentage of opened stoma in control was obviously decreased after 2 hours, whereas the percentage was increased in transgenic lines (Fig. 7C), implying that *SlUPA-like* promotes stomatal opening and decreases water retention under dehydration stress.

ABA is known for regulation of stomatal closure in response to abiotic stress and pathogen-associated molecular pattern (PAMP)-triggered immunity^39^. In order to discover the reason of stomatal behavior in transgenic lines, the expression levels of ABA biosynthetic, signal-transduction and responsive genes were monitored. *SlNCED1* was primarily involved in ABA biosynthesis in tomato^40^ and activation of SnRK2 by ABA can phosphorylate downstream effectors, leading to activation of ABA response^41^. In mature leaves of *SlUPA-L*-OE plants, expression of *SlNCED1* was remarkably repressed as well as *SlSnRK2.3*, implying that ABA biosynthesis and signal-transduction may be both suppressed (Fig. 7D). Meantime, three ABA responsive gene expressions, *CAT1*, *GME2* and *LEA*, were obviously repressed by 54–92% in transgenic plants, particularly *LEA*, indicating that ABA response is suppressed in transgenic plants (Fig. 7E)^42^,^43^,^44^. So, stimulating stomatal open in transgenic lines may be due to the inhibition of ABA response.

### Overexpression of *SlUPA-like* improves plant susceptibility to pathogens

**Figure 8:**
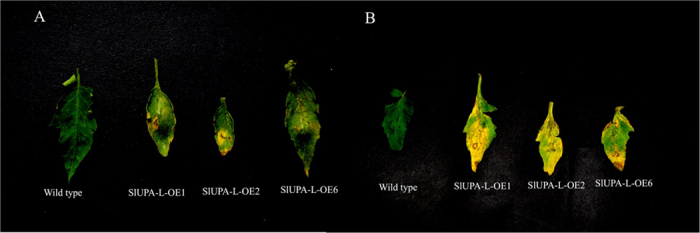
Leaflets from control and transgenic lines showed the phenotype after inoculation of *Xcc* for 3 days (**A**) and 8 days (**B**).

We have shown that GA response was excessively improved, instead JA response was repressed, and the percentages of opened stoma of *SlUPA-L*-OE plants were increased under stress condition. Given that GAs negatively regulate disease resistance of host plants^14^, and deficient JA level positively regulates the susceptibility of plant to *X. campestris*^45^, and stomatal opening provides access for infections of *Xcv*^46^, we logically speculate that *SlUPA-like* overexpressed plants may be susceptible to pathogens. To test this hypothesis, leaflets of transgenic and control plants were inoculated with *Xanthomonas campestris pv. campestris* (*Xcc*). As expected, the leaflets of transgenic lines showed yellow spot after 3 days of inoculation and spread out most parts of the blades after 8 days, whereas the control leaflets were unaffected all the time (Fig. 8). To further determine *Xcc* infection, a 253 bp DNA fragment from genome of *Xcc* was amplified in leaves of both AC^++^ and transgenic lines. The PCR products were only accumulated in transgenic lines (Fig. S7). Additionally, Fig. S8 showed that *SlUPA-L*-OE plants were more susceptible to plant disease than AC^++^ when exposed to infected plants. These data demonstrated that overexpression of *SlUPA-like* improves the susceptibility of tomato plants to pathogens.

## Discussion

GA-GID1-DELLA protein complex may contribute to the cell enlargement of young leaves. Hypertrophy development is attributed to cells enlargement along with decreased contents of starch^2^, which are similar to phenotypes of excessive GA accumulation. Loss-of-function of *eui* mutant shows elongated internode and reduced development of starch granule^47^. Experiment confirmed that overexpression of *SlUPA-like* induced cell enlargement and GA response (Figs 2 and 5). *LeKRP* genes were remarkably increased in both young and mature leaves of transgenic plants, together with the suppressed expressions of cell cycle related genes (Fig. 2), suggesting that overexpression of *SlUPA-like* facilitates the DNA endo-replication and inhibition of cell cycle. Meantime, both transcripts of *SlGID1-A* and *SlGID1-B* were significantly increased along with increased GA contents (Fig. 4C). Through phylogenetic analysis, AtGID1-b, SlGID1-A and SlGID1-B belong to the same clade (Fig. S5). *AtGID1b*-overexpressions can rescue GA response by forming the GA-GID1-DELLA protein complex^31^. In young leaves of transgenic plants, the key elements for formation of GA-GID1-DELLA protein complex coexisted. However, in mature leaves of transgenic lines, GA content were decreased (Fig. 5A), implying that this kind of protein complexes is probably destroyed. Therefore, the GA-GID1-DELLA protein complex probably contributes to the enhanced GA response and cell enlargement in young leaves of transgenic lines.

Expressions of *GAST1* are probably induced by overexpression of *SlPREs* independent of GA synthesis. In the young leaves of transgenic lines, expression levels of *SlPRE3* and *SlPRE4* were significantly increased compared to wide type (Fig. 4E). Similarly, transcripts of four *SlPREs*, *SlPRE1*-*SlPRE4*, were upregulated in the mature leaves and particularly the expression of *SlPRE4* was further magnified compared to young leaves (Fig. 5C). Meanwhile, *GAST1* expression was both increased in young and mature leaves, especially in mature leaves (Figs 4 and 5), indicating that expression of *GAST1* was sequentially induced by *SlPREs*. However, the expression of *EXP8* was suppressed in mature leaves (Fig. 5B), implying that the *GAST1* was regulated by different upstream signaling pathway. It is reported that *EXP8* does not respond to ABA and water, but specially responds to GA^30^. By contrast, expression of *GAST1* was response to both GA and ABA^29^. These results suggested that *GAST1* locates further downstream in GA signaling pathway. Moreover, it was proved that GA stimulates cell elongation through activation of *PREs*, which locate downstream point of GA biosynthesis^48^. Therefore, continuous GA response in transgenic lines was attributed to overexpression of *SlPRE*, which may be independent of GA biosynthesis.

Repressed JA response may not result in female sterility of flowers in transgenic lines. Plant protease inhibitors (PIs) can be regarded as defensive proteins to minimize the adverse effects from the attack of phytophagous insects or pathogens. In our experiment, the expression of *PI-I*, JA induced gene, was strongly repressed in *SlUPA-like*-overexpressed lines (Fig. 6C), suggesting that JA response is suppressed. However, the expression of *PI-II* was unaffected by overexpression of *SlUPA-like* (Fig. 6C), although its expression can be induced by JA^49^. Therefore, it need further exploration that JA response can be monitored by *PI-I* expression.

*jasmonic acid–insensitive1*(*jai1*) mutant is defective in JA signaling of tomato, exhibiting female sterility^50^. *jai1* mutants can set up fruit upon pollination, although the stigmas of flowers protrude from the anther cone during flower development^50^. That is, the anther filaments are not capable of reaching the position above the stigma at the time of flower blooming^51^. However, flowers in *SlUPA-L*-OE lines were sterile and their phenotypes were not similar to *jai1* (Fig. 3E). Moreover, the sterile flowers of transgenic lines didn’t have the ability to parthenocarpy when treated with 2, 4-D (data not shown). The reason of infertility of *SlUPA-L*-OE lines needs further research.

*SlUPA-like* may directly regulate the expressions of *SlJAZs. SlGIDs*, *SlPREs*, *SlJAZs* and *GAST1* were extremely induced by *SlUPA-like* (Figs 4, 5, 6). It was hypothesis that part or all of these genes contain the binding site for bHLH transcription factors within their promoters. The conserved recognition sequence of bHLH family is CANNTG (where N can be any nucleotide), in which the canonical sequence is CACGTG motif^52^. Within 1.5 kb upstream of the initiator-ATG, the numbers of putative binding site for bHLH were counted (Table S1). The numbers of binding sites in promoters of *SlGIDs* and *SlPREs* were not accordance with their expression levels, indicating that overexpression of two *SlGIDs* and four *SlPREs* may be indirectly regulated by SlUPA-like. The expression patterns of three *SlJAZs* (*SlJAZ2*, *SlJAZ10*, and *SlJAZ11*) largely accorded with the distribution of binding sites in their promoters (Table S1), revealing that these genes may be direct targets of SlUPA-like. Besides, the expression of *SlJAZ9*, homolog to *SlJAZ11*, is not induced by *SlUPA-like*. Previous works proved that G-rich DNA element is capable of formatting secondary structure and functions as repressor elements^53^,^54^. Within the promoter of *SlJAZ9*, Poly (G) motif from −1453 to −1733 bp makes it hard to be bonded by transcription factors. In addition, the promoter of *GAST1* contained no classical binding site, implied that excessive expression of *GAST1* may be mediated by other factors. Nevertheless, all these results from bioinformatics analysis were not sufficient to make conclusion, which need to be validated by chromatin immunoprecitation.

*SlUPA-like* may participate in the crosstalk between GA and JA response. In transgenic plants, expression levels of three *SlJAZs* were extremely up-regulated in transgenic leaves along with repressed JA response (Fig. 6). Meantime, *SlPREs* were significantly induced in transgenic plants with the activation of GA response. These data suggested that *SlUPA-like* may mediate the crosstalk of GA and JA response. Overexpression of *AtJAZs* has GA-hypersensitivity phenotypes, particularly *AtJAZ9* whose overaccumulation could be resistant to exogenous paclobutrazol^55^. In our research, extremely upregulated expressions of *SlJAZ9/10* (homologous to *AtJAZ7*/*AtJAZ8*)^38^ were found in transgenic plants. Moreover, overexpression of *SlPREs* shows GA response (Figs 4 and 5). So, stimulated GA response in transgenic plant may be due to the over-accumulation of both JAZ and PRE proteins. It was reported that JAZ9 interferes with DELLA-PIF3 interactions and competes with DELLA targets^55^. So, overexpression of *SlJAZ9/10* may destroy the inhibitory effect of DELLA proteins. However, DELLA proteins don’t interact with AtJAZ2, AtJAZ7 and AtJAZ8 *in vitro*^55^. So, whether the interactions between three SlJAZs and DELLA proteins mediated by other factors still needs to be tested. In addition, ILI1 and PRE1 inactivate the inhibitory function of IBH1 (another bHLH protein) through hetero-dimerization^56^. Therefore, it was possible that SlUPA-like protein is another target of PREs in tomato.

Stimulating stomatal opening may be mediated by ABA response in transgenic lines. Locating the epidermis of leaves, stoma is primary organ to lose water during transpiration and is also the key channel for pathogen infection. Plant have evolved stomatal defense to fight against the infection of pathogens. Certainly, plant pathogens secreted toxin or effectors to counteract stomatal defense to promote the stomatal reopening^16^. In this assay, when treated with severe water loss, most of stomatal pores closed in control plant, whereas more stoma in transgenic plants reopened (Fig. 7C). Meanwhile, expressions of ABA biosynthetic, signal-transduction and responsive genes were significantly suppressed in transgenic lines (Fig. 7D,E). To some extent, the stomatal behavior under infection of pathogen could be reproduced through dehydration treatments^16^. Thus, stimulated stomatal opening by overexpression of *SlUPA-like* may be mediated by suppressed ABA response.

Aberrant development facilitates the infections of pathogens in transgenic lines. Increased GA response, suppressed JA response and stimulated stomatal opening give us clues why overexpression of *SlUPA-like* leads to aberrant development (including curled and wilted leaves, sterile flowers and so on). It was reported that alterations of hormonal pathways improved plants susceptibility to *Xanthomonas*, which infection was mediated by stomatal behaviors^16^. Plant pathogens firstly gain entry into plant tissue via wounds or natural openings such as stomata. In order to counteract stomatal defense of host plant, pathogens can evade host immunity by suppressing the stomatal closure^16^. For these reasons, stomatal opening promoted by overexpression of *SlUPA-like* together with reprogrammed GA and JA response enhanced infection efficiency (Fig. 8), although the *Xcc* is not specific pathogen for *solanaceous* plants. This study revealed that *SlUPA-like* can be regard as a landmark gene in monitoring the disease tolerance of plants.

## Methods

### Plant Materials and Growth Conditions

In our experiment, *Solanum lycopersicum* Mill. cv. Ailsa Craig (AC^++^), a near-isogenic tomato line, was used as model plant grown in greenhouse (16-h-day/8-h-night cycle, 25 °C/18 °C day/night temperature, 80% humidity, and 250 μmol m^−2^s^−2^ light intensity). Plants of the first generation (T0) came from tissue culture. Tissue culture plantlets from transgenic lines and control with no construct were planted in greenhouse. The transgenic and control plants were synchronously planted in greenhouse. When the height of control plants reached 20–30 cm, the young leaves from middle part of plant were collected. Each line included 3–5 plants. When control plants set up fruits, mature leaves of 8 independent transgenic lines and control were harvested from top, middle and low part of each plant, and immediately frozen with liquid nitrogen, mixed, and stored at −80 °C until RNA extraction. The statistical analyses were performed. Each line included equal or greater than three plants.

### Sequence, structure and phylogenetic analysis

The exons and introns of genomic DNA were analyzed by GENESCAN (http://genes.mit.edu/GENSCAN.html). The theoretical molecular weight was calculated with the ExPASy compute Mw tool (http://expasy.org/tools/protparam.html). Multiple sequence alignment was conducted using the ClustalX 1.81 and DNAMAN 5.2.2 programs.

### Plant treatments

(a). Phytohormones treatments. 35-day-old AC^++^ seedlings were used for treatments. Tomato seedlings were sprayed with different concentration of phytohormones (50 uM IAA, 50 uM GA3, 50 uM MeJA, 100 uM ABA and 50 uM ACC)^57^ respectively until the water dripped, and then immediately enclosed in transparent bags with some small holes (about 0.5 cm in diameter) on the surface. After treatments for 0, 1, 2, 4, 8, 12 and 24 hours, the leaves of seedlings were harvested for analysis. All the samples were immediately frozen and stored at −80 °C until RNA extraction. Each treatment repeated with two times. (b). Dehydration treatments. 35-day-old AC^++^ seedlings were carefully pulled out from soil, washed with water and left on a piece of dry filter paper with 60% humidity at 25 ± 1 °C. After 0, 1, 2, 4, 8, 12, 24, 48 and 72 hours, the leaves were collected, frozen in liquid nitrogen and stored at −80 °C. The transgenic plants and wild type were planted in greenhouse and each line contains 3–5 plants. When the control set up fruits, mature leaves of transgenic lines and control were harvested from top, middle and lower part of each plant, and left on a piece of dry filter paper with 60% humidity at 25 ± 1 °C. After different hours, the statistical analysis of opened stoma in mature leaves was performed. (c). Mechanical wounding treatments. 35-day-old AC^++^ seedlings were used for mechanical wounding treatment. The leaflets were cut with scalpels into small pieces and left on a piece of wetted filter paper in sealed pots for 0, 1, 2, 4, 8, 12 and 24 hours, then the pieces of leaves were collected, respectively.

### Construction of *SlUPA-like* overexpression vector and plant transformation

Total RNA of AC^++^ leaf was extracted using Trizol (Invitrogen) according to the manufacturer’s instructions. Then, 0.5 μg of total RNA was used to synthesize first-strand cDNA through reverse transcription-PCR using Moloney murine leukemia virus reverse transcriptase (Takara) with tailed oligo(dT)_18_ primer. 2 μL of cDNA was used to clone the full-length cDNA with primers of *SlUPA-like*-F and dT-R through high-fidelity PCR (Prime START mix DNA polymerase; Takara). The amplified products were tailed by using the DNA-Tailing kit (Takara) and linked with pMD18-T vector (Takara). Positive clones were picked out via *Escherichia coli* transformation and confirmed by sequencing (Invitrogen). Then, a 993 bp specific DNA fragment of *SlUPA-like* was amplified with primers *SlUPA-like*-F and *SlUPA-like*-R, which had been tailed with *Bam*HI and *Sac*I restriction sites at the 5′ end, and linked into the pBI121 plasmid in the sense orientation to form RNA expression unit. Subsequently, the generated binary plasmid was transformed into *Agrobacterium tumefaciens* LBA4404 strain, following the previously described protocols^58^. Positive transgenic plants were selected on kanamycin medium and screened by polymerase chain reaction analysis with primers NPTII-F and NPTII-R. Then, T0 plants from each transgenic line were propagated through apex culture *in vitro* propagation.

### The percentages of open stomata under dehydration stress

Adult plants of AC^++^ and *SlUPA-like* overexpressed lines were planted in field. At 10:00 am, the mature leaves were sampled at the same position from the apex of compound leaves and immediately abaxial stomata of detached leaves were inspected with microscopy. The percentage of open stomata was calculated. Then, the detached leaves were placed in wetted filter paper. After 1, 2, 3 and 4 hours of dehydration treatments, the percentage of opened stomata was calculated, respectively. All experiments were repeated 3 to 5 times.

### The root growth inhibition by MeJA

The aseptic axirllay buds with approximately same size from AC^++^ and transgenic lines were planted in rooting MS medium. The different concentration of methyl jasmonate (MeJA), 0 uM, 10 uM, 20 uM and 50 uM, was added into rooting medium, respectively. After 25 days of cultivation, the lengths of roots were calculated. Three biological replicates were used for each treatment.

### Quantitative real-time PCR analysis

Total RNAs from different tissues of AC^++^ and transgenic lines were extracted. Quantitative real-time PCR was performed using the SYBR Premix Ex Taq II kit (Promega) in a 10 μL total sample volume (5.0 μL of 2 × SYBR Premix Ex Taq, 0.5 μL of primers, 1.0 μL of cDNA, and 3.5 μL of deionized water) under the following conditions: 95 °C for 2 min, followed by 40 cycles of 98 °C for 15 s and annealing temperature for 30 s. To remove the interference of genomic DNA and the template from the environment, no-template control and no-reverse transcription control experiments were performed. Additionally, three replications for each sample were used, and standard curves were run simultaneously. The tomato *SlCAC* and *SlEF1a* genes were used as internal standards^59^. Primers used for quantitative RT-PCR are shown in supplementary Table S2.

### Microscopy

The detached leaves sampled from AC^++^ and transgenic lines were immediately fixed by formalin–acetic acid–alcohol fixative (FAA). Then, steps of dehydration, fixation, sectioning and dewaxing were operated on the fixed materials. Finally, the treated materials were observed under the microscope.

### Chlorophyll quantification in tomato leaves

For chlorophyll measurement, leaves were sampled from control and transgenic plants at the same height and simultaneously weighed. Samples were grinded into powder with liquid nitrogen, extracted with 10 mL 80% aqueous acetone (v/v). The extracts were centrifuged at 4, 000 g for 5 min and the absorbances of the supernatant were measured at 646 and 663 nm, using a lambda 900 scanning spectrophotometer (PerkinElmer). Total chlorophyll contents were calculated by the following equation: Chl (mg/mL) = 20.29(OD645) + 8.02(OD663), Chla (mg/mL) = 12.7(OD663)−2.69(OD645), and Chlb (mg/mL) = 22.9 (OD645)−4.677 (OD663). OD represents the absorbance, while 645 and 663 represent the wavelength. Chl, Chla and Chlb represent total chlorophyll, chlorophyll a and chlorophyll b, respectively.

### Quantification of Gas

In this assay, quantity of endogenous GAs was determined by GA ELISA Kit (Sangon Biotech, Shanghai, China). The fresh samples including young and mature leaves from AC^++^ and *SlUPA-L*-OE lines were grinded in liquid nitrogen. About 1 g treated sample was extracted with 80% methanol and centrifuged at 1000 g for 15 min. The supernatant was partitioned against ethyl acetate and purified by Sephadex chromatography and C18 cartridges^60^. Standards or samples were then added to the appropriate microtiter plate wells with a biotin-conjugated polyclonal antibody preparation specific for GA and Avidin conjugated to Horseradish Peroxidase (HRP) was added to each microplate well and incubated. Then a TMB substrate solution was added to each well. Only those wells that contain GA, biotin-conjugated antibody and enzyme-conjugated Avidin will exhibit a change in color. The enzyme-substrate reaction was terminated by the addition of a sulphuric acid solution and the color change was measured spectrophotometrically at a wavelength of 450 nm ± 2 nm by Multimode Reader. The concentration of GA in the samples was determined from the standard curve.

### Infection experiments

In consideration of infectivity of *Xanthomonas campestris pv. campestris (Xcc)*, mechanical damages were caused on leaflets of AC^++^ and transgenic lines by toothpick. Immediately, 1 *ul* inoculums were placed into wounds. Then, the leaflets were sealed by one transparent plastic bag. A few days later, bacterial growth in leaflets was monitored. After 3 and 8 days of inoculation, the inoculated leaves were sampled for DNA extraction by CTAB. A 253-bp genomic DNA fragment of *Xcc* was amplified with primers (Table S2). A 253-bp fragment of M. oryzae 28S rDNA was amplified from fungal genomic DNA using the primers 5′–TCGCCTACCGAGAAATCCC–3′ and 5′–CCGAACCCTTGACCAGCAT–3′, to monitor the infections of *Xcc* and the products were detected by agarose gel electrophoresis.

## Additional Information

**How to cite this article**: Cui, B. *et al.* Overexpression of *SlUPA-like* induces cell enlargement, aberrant development and low stress tolerance through phytohormonal pathways in tomato. *Sci. Rep.* **6**, 23818; doi: 10.1038/srep23818 (2016).

## Supplementary Material

Supplementary Information
