# Supplementary material for: Overexpression of SlUPA-like induces cell enlargement, aberrant development and low stress tolerance through phytohormonal pathway in tomato
Source: Sci Rep. 2016 Mar 30;6:23818. doi: 10.1038/srep23818 (PMC4812305; doi:10.1038/srep23818)

Title:

Overexpression of *SlUPA-like* induces cell enlargement, aberrant development and low stress tolerance through phytohormonal pathway in tomato

Authors:

Baolu Cui, Zongli Hu, Jingtao Hu, Yanjie Zhang, Wencheng Yin, Zhiguo Zhu, Ye Feng, and Guoping Chen *

Key Laboratory of Biorheological Science and Technology (Chongqing University), Ministry of Education, Bioengineering College, Chongqing University, Chongqing 400044, People's Republic of China

Supplementary Information

Table S1. Statistical analysis of putative binding sites for bHLH transcription factor in the promoters of related genes

Genes	the number of CANNTG motifs	the number of CACGTG motifs	Chromosomes
SlGID1-A	3	1	9
SlGID1-B	2	0	6
SlGID1-C	2	2	1
SlGAST1	6	0	2
SlPRE1	8	1	2
SlPRE2	3	0	2
SlPRE3	8	0	6
SlPRE4	6	0	5
SlPRE5	9	0	4
SlJAZ2	2	0	12
SlJAZ9	3	1	8
SlJAZ10	8	1	8
SlJAZ11	6	1	8

Table S2. PCR primers used in this study

Primer names	Sequences (5′ → 3′)
qSlCAC-F	CCTCCGTTGTGATGTAACTGG
qSlCAC-R	ATTGGTGGAAAGTAACATCATCG
oligo(dT)	GCTGTCAACGATACGCTACGTAACGGCATGACAGTGTTTTTTTTTTTTTTTTT
qNPTII-F	GACAATCGGCTGCTCTGA
qNPTII-R	AACTCCAGCATGAGATCC
SlUPA-like-F	CGCGGATCCATGGCTGCTTTTTCATCACACC
SlUPA-like-R	CCGGAATTCTTAATGGAAAGAACAAAAGTTGTTGC
dT-R	GCTGTCAACGATACGCTACGTAACG
qSlUPA-like-F	GGGTCTTCTTCTATGACTTCTGCT
qSlUPA-like-R	TTTCATCTTTGGCTACTAACTTGC
qSlEF1α-F	TACTGGTGGTTTTGAAGCTG
qSlEF1α-R	AACTTCCTTCACGATTTCATCATA
qGA20ox1-F	TTCTCAAATTGGCTTCATGATCAA
qGA20ox1-R	TTCCCCCTAATTCCCATAACAT
qGA20ox2-F	TAAGAAGGATAAGGTGGTGAGGC
qGA20ox2-R	CCGTAGTTTTCTGTTGAAGCCA
qGA3ox1-F	ATAGGCACCCACCCTTGTATA
qGA3ox1-R	GGATGAAAGTGCCTTGTCAAAAT
qGA3ox2-F	GTAGACCAAAGGAACCCTCAAAT
qGA3ox2-R	GCCGAACAGATGAAAGTGCT
qGA2ox1-F	ATTAAGATCCAATAACACTTCG
qGA2ox1-R	TCTTGATTTCACACTATTTGC
qGAST1-F	CAACAACAGAGAAATAACCAAC
qGAST1-R	TTATACGATGTCTTTGAACACC
qSlPRE1-F	CGAAAGAACGAAAAGAGAGACATT
qSlPRE1-R	GCTAGAGCGACGATTGCGAA
qSlPRE2-F	TATGTCTGGGAGAAGGTCAAGGA
qSlPRE2-R	CGACGATTACGAATTTCAGGAAG
qSlPRE3-F	TTCACACTCTCCATAGCAACACAT
qSlPRE3-R	TCCTCACTTATCCTTGATCCTCC
qSlPRE4-F	ATCAAATCGCTGATCTTGTTTCC
qSlPRE4-R	CACTTAATCCATCCACTTCTCTGTGTA
qSlPRE5-F	CCTTCTCTCTTTGTCCATAACTTGTC
qSlPRE5-R	CCTGATGATTGTCTCGAACCG
qSlExPA8-F	CTGATTTGTTAAATGTTCGGTGA
qSlExPA8-R	AAGAATGATTCTTTTTTTGGGGA
qKRP1-F	GCGGTGATGGTGGGTCGTAT
qKRP1-R	CGTCCTTAACACCCTTTCCCTC
qKRP2-F	TTCACAAACCACCCACCCC
qKRP2-R	TGACCATTTCGTCCACCTCC
qKRP3-F	GGCTGGAGAAACCCTTGG
qKRP3-R	CCCTCAAACTCCGATTCTGTC

qKRP4-F	CACAAGGAAGAGGAAGAAGCG
qKRP4-R	CCAAAACCAGATGCTGAAACG
CycA3;1-F	CTAAGAAAAGAGCAGCAGAAGCA
CycA3;1-R	GATTCCTTATCTTTTTCAGCAACAG
CycB1;1-F	GTATCTCGCCCCGTAACAAG
CycB1;1-R	TCTCCTCAGGTTTTGGCTTT
CycD2;1-F	CTGCCAAAGCCTCAAGCG
CycD2;1-R	CAGTGGAGCTAGTGTCATTCGC
qSlJAZ2-F	TAGCCAACAAACAGAACCCCA
qSlJAZ2-R	AAGTGAATTCCGTCTCGCGAT
qSlJAZ10-F	GGAACTCACTCTTTCTCCTAGCAAC
qSlJAZ10-R	TGGTGATGAAGGCTCAGACAGCTT
qSlJAZ11-F	GGAGTTTAGGCTTATGCCACCTTC
qSlJAZ11-R	GGCTCAGATATTGGTGACAGACTC
qSlGID1-A-F	GCGGTGTTGTTGAATGAGAATC
qSlGID1-A-R	GTCTTGTGCAGATCAGCTCCC
qSlGID1-B-F	GGTGTTTATTCATTTGATGTTGTTG
qSlGID1-B-R	AGAATGTGTCGTAAATAGCACTGTT
qSlGID1-C-F	GCCTACTTAGCCGTGTCTATCG
qSlGID1-C-R	AGTGTGCAAAACTTCCACCATG
SlUPA-like-F	GGGTCTTCTTCTATGACTTCTGCT
SlUPA-like-R	TTTCATCTTTGGCTACTAACTTGC
qSlPI-I-F	CTTCTTGCAACTTCCTTTG
qSlPI-I-R	TGTTTTCCTTCGCACATC
qSlPI-II-F	ATGCTTGCACCTTTAATTGTGATC
qSlPI-II-R	TAATAGCAACCCTTGTACCCTGTG
qSlNCED1-F	CCCGATTTGGTATTCTGGATAAGTA
qSlNCED1-R	GAGACGGATTTCGGATAAAACACT
qSnRK2.3-F	ATCATTACCTCACTGGAAGCTTGGAC
qSnRK2.3-R	AAACAGTGGATACCAAAAGATCGCC
CAT1-F	AAATGGGTTGAGTCTTTATCCGA
CAT1-R	TCATTGATTTTTCACATTGTAGGCT
GME2-F	CCATCACATTCCAGGACCAGA
GME2-R	CGTAATCCTCAACCCATCCTTC
LEA-F	TATTGGTAAAGATTGGGACATTGA
LEA-R	TGTCTTCTTGTTTGTCACCGTTC
Xcc-F	TCGCCTACCGAGAAATCCC

Figure S1 | Adaxial and abaxial pavement cell of leaves were detected by hand-sliced method and magnification was 40×. The adaxial epidermal cell of AC^{++} (A) and *SlUPA*-*L*-OE lines (B). The abaxial epidermal cell of AC^{++} (C) and *SlUPA*-*L*-OE lines (D).

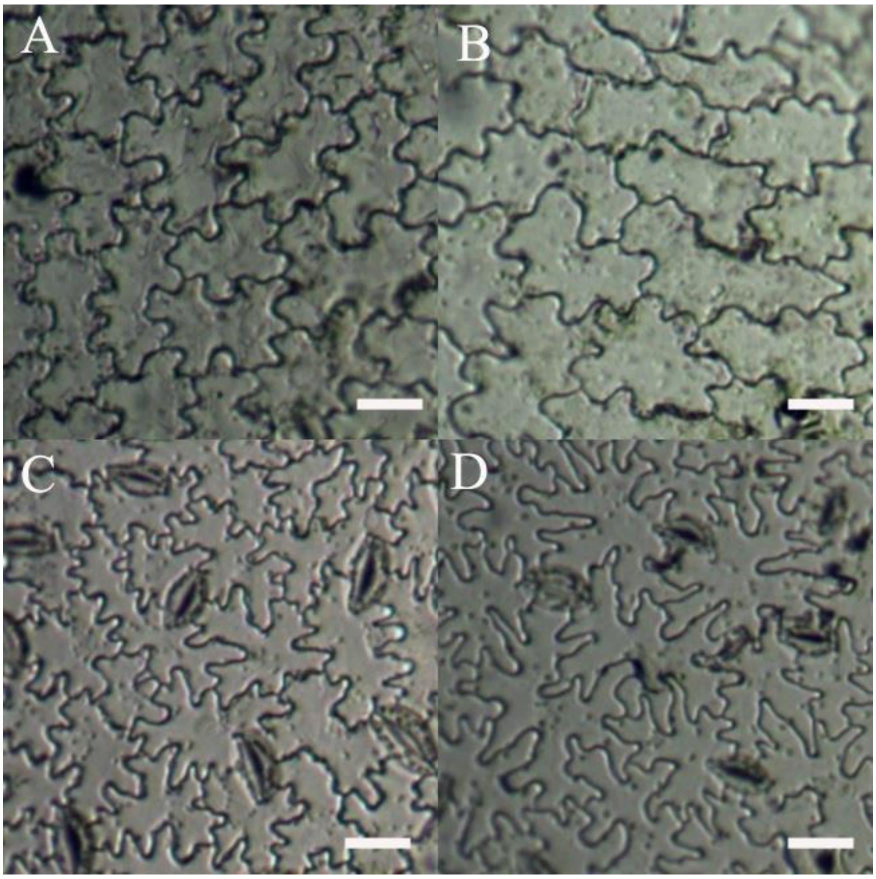

Figure S2 | Phylogenetic analysis of *KRPs* in tomato and Arabidopsis.

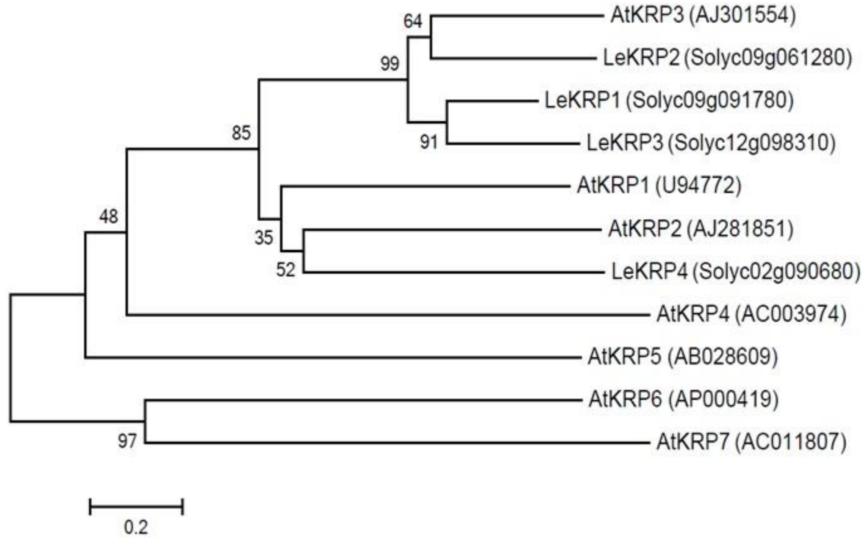

Figure S3 | The leaf veins displayed different phenotypes in control and *SlUPA-L*-OE lines. The phenotypes of leaf vein from AC++ (A) and of transgenic lines (B). Their magnification was 40×. (C). The phenotypes of leaf vein of AC++ and the magnification was 80×. PM, Phloem; XM, Xylem; PT, Palisade Tissues; ST, Spongy Tissues; PC, Parenchyma Cells.

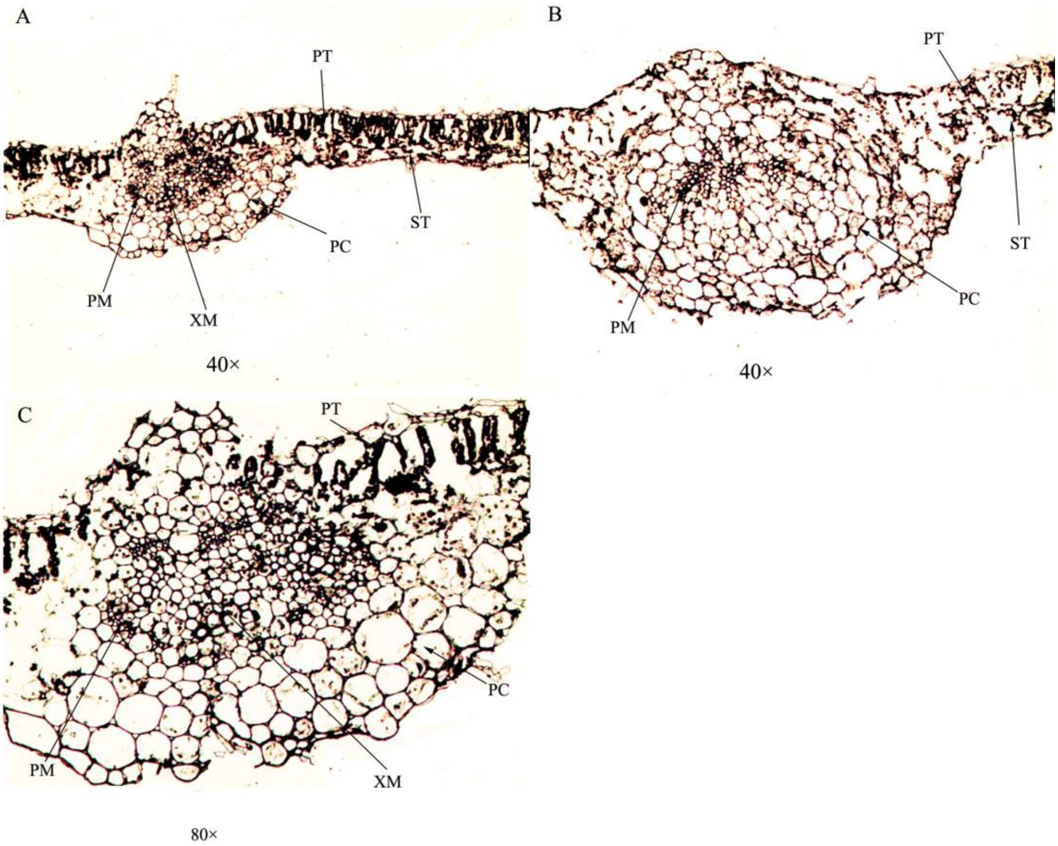

Figure S4 | The auxiliary buds further from apex of plants was repressed in *SlUPA-L*-OE lines.

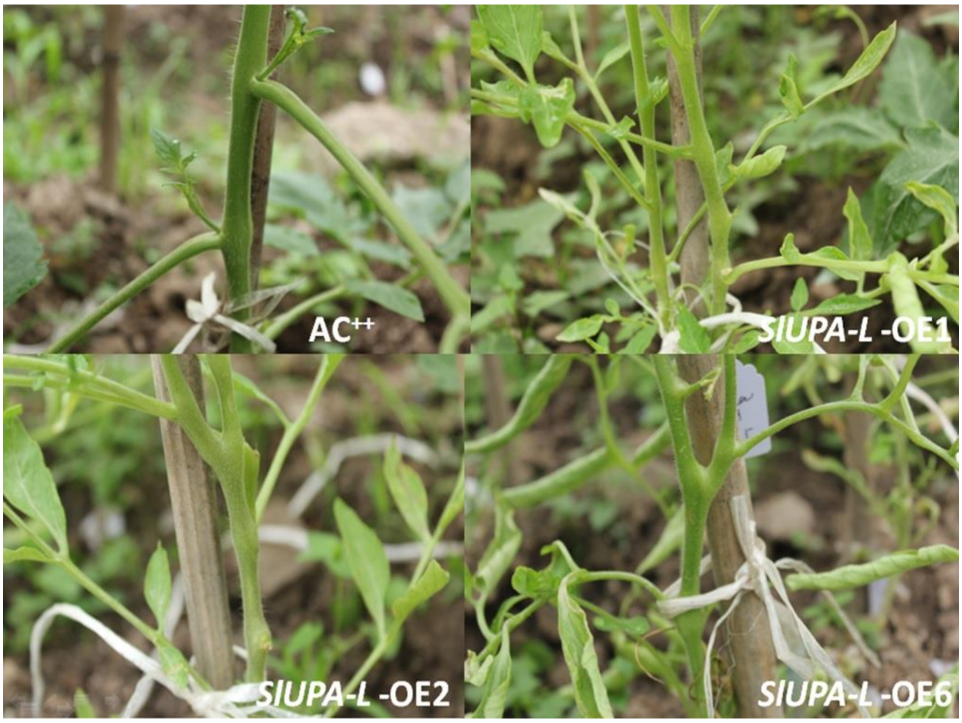

Figure S5 | Phylogenetic analysis of *GID1s* genes in Arabidopsis and tomato.

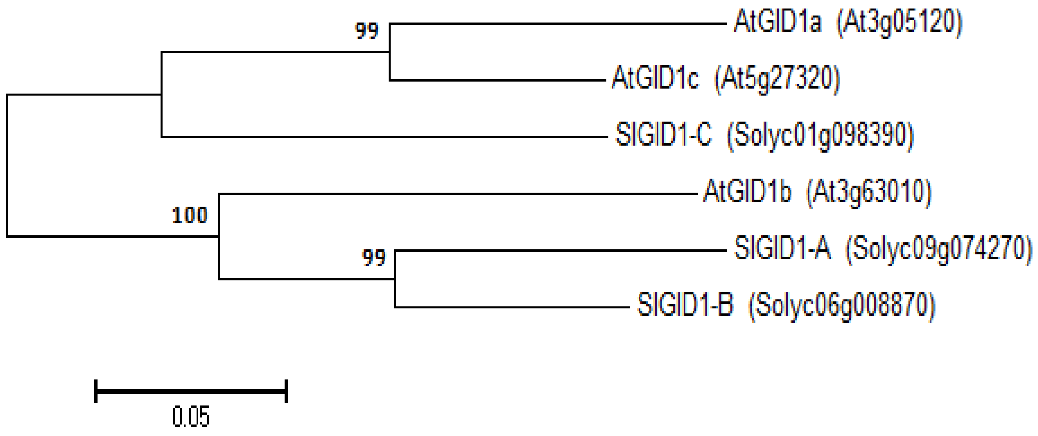

Figure S6 | Phylogenetic analysis of *PREs* genes in Arabidopsis and tomato.

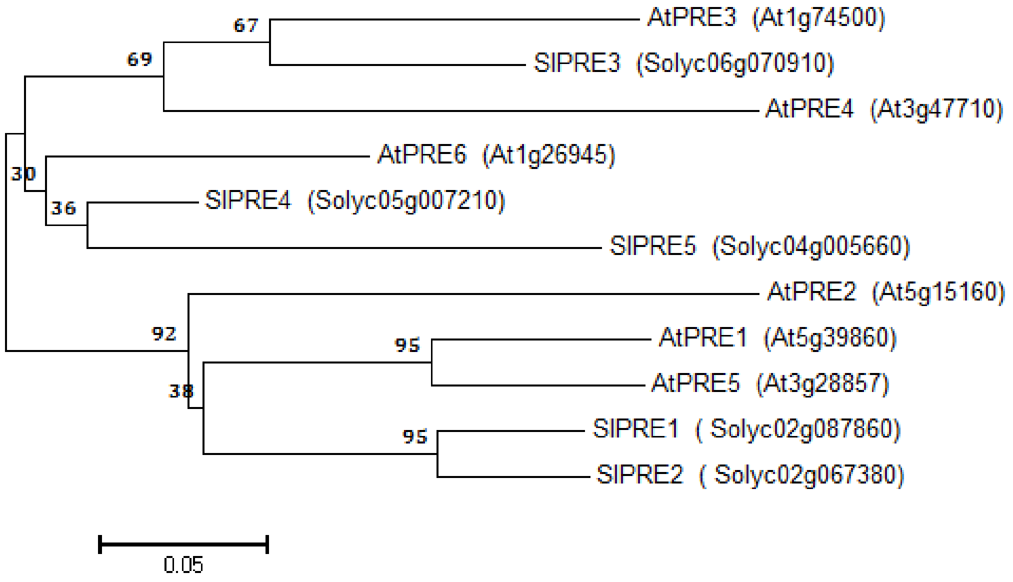

Figure S7 | Ddetection of PCR products of *Xcc* genomes after 8 days post-inoculation by agarose gel electrophoresis.

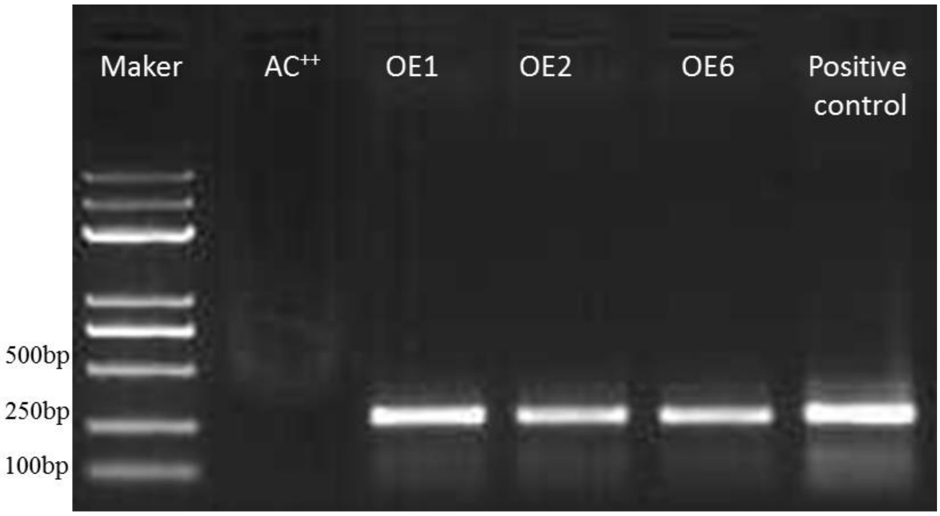

Figure S8 | AC^{++} and *SlUPA-L*-OE1 showed sick phenotypes when exposed to disease plants after 10 days. The phenotypes of AC^{++} not exposed to disease plants (A) and exposed to disease plants (B) for 10 days. The phenotypes of *SlUPA-L*-OE1 not exposed to disease plants (C) and exposed to disease plants (D) for 10 days.

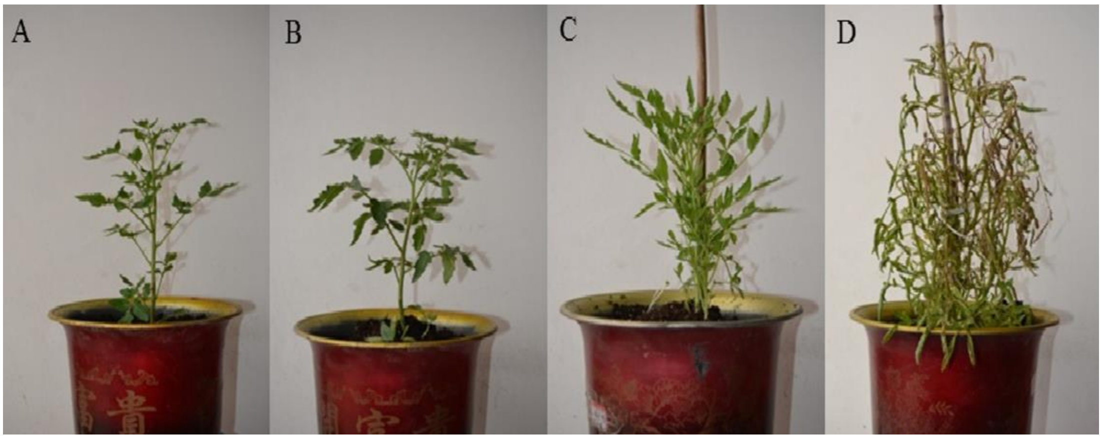

Supplement: Supplementary Information [file srep23818-s1.pdf]
